# Estimating population immunity to SARS-CoV-2 by random sampling from primary and secondary healthcare in Scotland, May 2024

**DOI:** 10.1016/j.ebiom.2025.105760

**Published:** 2025-05-16

**Authors:** Mhairi J. McCormack, Sam Scott, Nicola Logan, Savitha Raveendran, Joseph Newman, Igor A. Santos, Dalan Bailey, Pablo R. Murcia, Emma C. Thomson, Margaret J. Hosie, Brian J. Willett

**Affiliations:** a MRC University of Glasgow Centre for Virus Research, Garscube Estate, Glasgow, United Kingdom; b The Pirbright Institute, Guildford, Surrey, United Kingdom

**Keywords:** COVID-19, Vaccine, Neutralization, IgG4, Variant

## Abstract

**Background:**

As the COVID-19 pandemic has ended, the global focus has shifted from “pandemic response” to “long-term management”. With no ongoing nationwide serosurveillance studies, our understanding of the level of immunity in the general population has diminished. In this study, we screened random samples from a biorepository serving the largest health board in Scotland for antibodies against SARS-CoV-2 to define the current immunological landscape, informing vaccine strategies going forward.

**Methods:**

997 pseudonymized serum samples were obtained from NHS Greater Glasgow and Clyde (NHS GGC) biorepository in May 2024, along with associated data for age, sex, and COVID-19 vaccine history. Samples spanned ages from 19 to 98 years, with 59.0% female and 41.0% male, and 39.1% from primary healthcare (GP practices) and 61.0% from secondary healthcare (hospitals). Anti-SARS-CoV-2 receptor binding domain (RBD)-specific antibodies were measured by enzyme-linked immunosorbent assay (ELISA), while neutralising antibodies were quantified using HIV(SARS-CoV-2) pseudotype-based virus neutralisation assay (PVNA). ELISAs measured both total IgG and IgG4-mediated responses. Pseudotypes were prepared bearing spike proteins from vaccine antigens B.1 and XBB.1.5, contemporaneous circulating variants KP.3.1.1 and LB.1, and the emerging variant XEC. Samples were grouped by number of COVID-19 vaccine doses received (from no vaccination to ≥8 doses) and 12 samples from each group were screened by ELISA and PVNA.

**Findings:**

The random selection of 1000 samples provided a broad cross-section of the population derived from patients with a range of individual vaccine histories, from those having received no COVID-19 vaccines to those having received 8 or more doses. The number of doses received increased with age, from a mean age of ∼40 for those having received one dose to a mean age of 77–78 for those having received 7 or 8 doses. While total IgG responses were similar across each of the groups, irrespective of vaccine history, repeated exposure to mRNA-based vaccines elicited an increase in SARS-CoV-2-specific IgG4. Neutralising antibody titres against the vaccine antigens B.1 and XBB.1.5 increased with age, reaching maximum geometric mean titres of 5610 (95% CI, 2773–11,349) for B.1 and 4577 (1832–11,440) for XBB.1.5 in those receiving 8 doses. In all groups, titres measured against the KP.3.1.1, LB.1 and XEC were significantly lower, consistent with the emergence of immune evasive variants over time. Cross-neutralisation of KP.3.1.1 was limited to maxima of 145 (62.2–336) and 187 (83.8–418) in the 7 and 8 dose groups, while titres against XEC were 105 (47–233) and 90.9 (48.1–172) respectively.

**Interpretation:**

In the absence of systematic COVID-19 serosurveillance, random sampling of sera from biorepositories associated with major health boards can generate valuable data about the level of immunity in the general population, informing estimates of vaccine effectiveness and antigen selection.

**Funding:**

United Kingdom 10.13039/501100000265Medical Research Council and Genotype-to-Phenotype National Virology Consortium.

**Research in context**

**Evidence before this study**

We searched PUBMED from database inception to February 12th, 2025, using the keywords (“SARS-CoV-2” OR “COVID-19” OR “COVID”) AND (“neutralizing antibody” OR “neutralising antibody”) AND “surveillance” AND (“2024” or “2025”), returning twenty-three results. The majority of the studies retrieved, examine the nature of the immunity elicited by vaccination or infection, and the level of neutralising activity against known variants. No studies were found that described the current level of immunity in the general population and how future surveillance could be conducted in a rapid and cost-effective way.

**Added value of this study**

We took advantage of a readily available source of samples (the biorepository associated with a general hospital serving a major city on the United Kingdom (Glasgow), and sample metadata (the NHS Greater Glasgow and Clyde Safe Haven), to assess the level of immunity in general population. At a very modest cost, we were able to show that the sample set covered patients with a broad range of ages, and vaccine histories, from no vaccination through to eight or more doses of vaccine. From this sample set, we were able to demonstrate the effects of age, number of doses received, and waning immunity on the level of neutralising activity against current SARS-CoV-2 variants.

**Implications of all the available evidence**

With the ending of nationwide serosurveillance studies, our understanding of the level of immunity in the general population has diminished markedly. This study demonstrates that analysis of a random selection of residual samples from biochemistry laboratories serving hospitals of major cities can yield valuable data on levels of immunity in the general population, informing the selection of vaccine antigens and strategies for protecting susceptible communities from COVID-19.

## Introduction

Following the emergence of SARS-CoV-2 in December 2019, the global spread of the virus was relentless, resulting in an estimated ∼777 million cases and ∼7 million deaths (WHO^1^). The development of safe and effective vaccines played a significant role in reducing morbidity and mortality, reducing hospitalisation and the likelihood of infections requiring intensive care.^2^ Four years into the COVID-19 pandemic, the immunological landscape is considerably more complex, as populations have been exposed to successive waves of distinct viral variants and types of vaccine.

Since the start of the COVID-19 pandemic, the United Kingdom experienced several epidemic waves associated with the emergence of SARS-CoV-2 variants. The ancestral Wuhan-Hu-1-like virus, and its subsequent D614G mutant (lineage B.1), were followed by the Alpha (B.1.1.7), Delta (B.1.617.2; “AY.X”) and Omicron (B.1.1.529; “BA.X”).^3^ More recent variants stem from the BA.2.86 lineage,^4^ giving rise to the variant JN.1^5^ and derivatives including KP.3.1.1^6^ and recombinant viruses such as XEC.^7^ Similarly, while the original SARS-CoV-2 vaccines (ChAdOx1, BNT126b2, mRNA-1273),^8^, ^9^, ^10^, ^11^, ^12^, ^13^ introduced in the UK from December 2020 onwards, were based on the spike glycoprotein from the ancestral Wuhan-Hu-1, with minor modifications to enhance stability and accessibility of the endogenously-synthesised antigen, subsequent vaccines have incorporated antigens from successive viral variants; from the bivalent B.1/BA.1 (Comirnaty and Spikevax Bivalent)^14^^,^^15^ (Autumn 2022) and B.1/BA.4-5 (Comirnaty Original/Omicron BA.4-5 and Spikevax Bivalent Original/Omicron BA.4-5)^16^, ^17^, ^18^ vaccines (Spring 2022), to more recent monovalent iterations based on XBB.1.5^19^, ^20^, ^21^ (Comirnaty Omicron XBB.1.5 and Spikevax XBB.1.5) (Autumn 2023).

As the COVID-19 pandemic has progressed, the global focus has shifted from “pandemic response” to “long-term management”. Nationwide serosurveillance studies have now ended, and as a result, our understanding of the level of immunity in the general population has diminished. While vaccination campaigns have sought to maintain immunity in at-risk populations; the elderly, healthcare workers, and those with weakened immune systems, in the United Kingdom the majority of the population are no longer being offered COVID-19 vaccination. Current vaccination guidelines for Autumn 2024 recommend that booster vaccines should be given to adults aged 65 years and over, residents in care homes for older adults, and individuals aged 6 months and over in a clinical risk group.^22^ Hence, for the majority of the general population, immunity is now being elicited through exposure to successive waves of infection with SARS-CoV-2 variants as they circulate as endemic, seasonal coronaviruses. Accordingly, most people now have “hybrid immunity”, generated through a combination of vaccinations and infections. Moreover, evidence has emerged that repeated vaccination of at-risk populations such as the elderly with mRNA-based COVID-19 vaccines has resulted in a marked increase in the level of isotype class-switching to IgG4.^23^, ^24^, ^25^, ^26^ Understanding the complex immunological landscape of the general population will be crucial to formulating COVID-19 vaccination strategies going forward. Given the ending of nationwide serosurveillance studies, the aim of this study was to determine whether the level of immunity in the general population to current SARS-CoV-2 variants could be determined by random sampling of sera from the biorepository associated with a major health board.

## Methods

### Serum samples

Serum samples were obtained from the National Health Service Greater Glasgow and Clyde (NHSGGC) Biorepository, a resource for clinical research based at the Queen Elizabeth University Hospital (QEUH), Glasgow. For this study, random residual biochemistry serum samples (n = 997) in serum separator tubes (SST) from primary (general practices) and secondary (hospitals) health care settings were collected by the NHSGGC Biorepository between the 14th of May 2024 and 30th of May 2024. Associated metadata included age, sex, care type, date of sample collection, date of positive COVID-19 PCR results, date of COVID-19 vaccination, and vaccine manufacturer were retrieved from the NHSGGC Safe Haven. Full details of sample collection are in Supplementary Methods. For the experimental analysis included in this study, we tested a subset of the cohort (n = 108, see Supplementary Methods for additional information).

### Ethics

Ethical approval for the collection of residual samples from Clinical Biochemistry was provided by NHSGGC Biorepository (application 837).

### Enzyme linked immunosorbent assays (ELISAs)

ELISAs for SARS-CoV-2 antibodies were performed as described previously.^27^ Briefly, 96-well plates were coated overnight at 4 °C with purified SARS-CoV-2 (Wuhan-Hu-1) spike RBD antigen^27^ in phosphate-buffered saline (PBS). Wells were blocked for 1 h at room temperature in blocking buffer consisting of PBS with 0.05% Tween 20 (PBS/Tween) and 1× casein (Vector labs., Peterborough, UK). Plates were then washed 3× in PBS/Tween prior to incubation with 50 μL of each serum sample diluted 1:100 in blocking buffer. Each plate included two pooled negative controls and two pooled positive controls. Sera were incubated for 1 h at room temperature. Plates were then washed 3× with PBS/Tween, before incubation for 1 h with either horseradish peroxidase (HRP)-conjugated rabbit anti-human IgG H + L (Bethyl labs., via Cambridge Bioscience, Cambridge, UK) diluted 1:2500 in blocking buffer, or horseradish peroxidase (HRP)-conjugated mouse anti-human IgG4 Fc (Southern Biotech, via Cambridge Bioscience). Plates were washed a further 3× in PBS/Tween before addition of the 3,3′,5,5′-tetramethylbenzidine (TMB) liquid substrate (Sigma Aldrich, Merck, Dorset, UK). Colour development was allowed to proceed for 10 min before the addition of 1 M H_2_SO_4_ stop solution, at which point the absorbance was determined at 450 nm on a Multiskan FC plate reader (Thermo Fisher). Full validation of the RBD ELISA has been described previously.^27^

### Neutralisation assay

Neutralising activity was measured using a pseudotyped-virus neutralisation assay (PVNA) as described previously,^27^, ^28^, ^29^ in which the SARS-CoV-2 spike proteins are expressed on the surface of HIV particles bearing a luciferase marker gene. This assay format has been shown previously to display an excellent correlation with live virus assays for SARS-CoV-2 neutralising antibodies.^30^ HEK293 (RRID:CVCL_0045), HEK293T (RRID:CVCL_0063) and 293-ACE2 (MRC University of Glasgow Centre for Virus Research, Glasgow) cells were maintained in Dulbecco's modified Eagle's medium (DMEM) supplemented with 10% foetal bovine serum, 200 mM l-glutamine, 100 μg/ml streptomycin and 100 IU/ml penicillin. HEK293T cells were transfected with the appropriate SARS-CoV-2 spike gene expression vector in conjunction with lentiviral vectors p8.91^31^ and pCSFLW^32^ using polyethylenimine (PEI, Polysciences, Warrington, USA). Pseudotype-containing supernatants were harvested 48 h post-transfection, aliquoted and frozen at −80 °C prior to use. The B.1, XBB.1.5, KP.3.1.1, LB.1 and XEC spike expression constructs are described in Supplementary Methods. All cell culture media and supplements were obtained from Thermo Fisher Scientific, Paisley, Scotland).

293-ACE2 target cells were maintained in complete DMEM supplemented with 2 μg/ml puromycin. Neutralising activity in each sample was measured by a serial dilution approach. Each sample was serially diluted in triplicate from 1:50 to 1:36,450 in complete DMEM prior to incubation with approximately 1 × 10^6^ counts per second (CPS) per well of HIV (SARS-CoV-2) pseudotypes, incubated for 1 h, and plated onto 293-ACE2 target cells. Pooled purified human IgG (KIOVIG 10%, Lot: BE12C258AG, Takeda UK Ltd) was included in the analysis as a positive control for neutralising activity. Luciferase activity was quantified after 48–72 h by the addition of Steadylite Plus chemiluminescence substrate and analysis on a PerkinElmer EnSight multimode plate reader (PerkinElmer, Beaconsfield, UK). Antibody titre was then estimated by interpolating the point at which infectivity had been reduced to 50% of the value for the ‘no serum’ control samples in Microsoft Excel (see Supplementary Methods).

### Statistical analyses

997 residual biochemistry samples selected at random were obtained from individuals seeking primary or secondary healthcare. By 2023, in a fully vaccinated population, COVID-19 seroprevalence was estimated at 99.9% (95% CI 99.8%–99.9%) using the Roche S assay.^33^ Samples were stratified into groups based on number of vaccine doses received. A power analysis was then conducted to ascertain how many samples from each group we would need to test, to detect differences in geometric mean values between the dose groups while balancing cost and feasibility. We based our power calculation on our previous estimates of antibody titres against ancestral lineage (B.1) and Omicron lineages (BA.1 and BA.2.86) in a similarly vaccinated population.^34^ Given a sample size of n = 12 per group, a two-sample t-test on log-transformed values was used to assess statistical power at α = 0.05. The comparison between B.1 (GM = 1293.64) and BA.1 (GM = 205.24) yielded an effect size of Cohen's d = 1.36, with an estimated power of 0.89 (89%), indicating sufficient power to detect a statistically significant difference. The comparison between B.1 (GM = 1293.64) and BA.2.86 (GM = 61.89) resulted in an effect size of Cohen's d = 3.32, with an estimated power of 1.00 (100%), confirming a highly significant difference between these groups. These data suggested that a sample size of n = 12 per dose group would be adequate to detect meaningful differences in geometric mean titres against each variant across the groups.

Statistical analyses of the data were performed using Prism 8.4.3 software (GraphPad), Microsoft Excel, and in RStudio. Differences in ELISA absorbance between IgG and IgG4 were compared using the Mann–Whitney test for unpaired, non-parametric data. Comparisons of neutralising antibody titres against variants within age or dose groups used a One-Way ANOVA with Friedman's test, corrected for multiple comparisons using Dunn's test. To assess the correlation between “Age”, “Doses” and “Days since last vaccination”, we used the nonparametric Spearman's test (RStudio corr (data)). Anything >0.5 was considered highly correlated. The Generalized Additive Model (GAM) used to examine the relationship between antibody responses and “Age”, number of vaccine doses (“Doses”), time elapsed since last vaccination was administered (“Days since last vaccination”) was performed using the package mgcv in RStudio. For factor terms, statistical significance was determined using a Wald z-test, while for smooth and interaction terms, statistical significance was determined using a Chi-squared test based on a penalised likelihood ratio test with the penalisation applied using thin plate regression spline bases to control for overfitting. For additional detailed description of the statistical analyses see Supplementary Methods.

### Role of the funding source

The funders of the study had no role in the design of the study, data collection, data analysis, data interpretation, or in the writing of the report.

## Results

In the absence of ongoing, national-level community serosurveillance, we sought to gauge the level of immunity to SARS-CoV-2 in the general population by examining a series of ∼1000 random serum samples obtained from the NHSGGC Biorepository in May 2024 as part of an ongoing respiratory virus surveillance study. Serving a population of approximately 1.2 million in Greater Glasgow, we reasoned that such a cross-section of samples from the biorepository would provide a “snapshot” of the immunological landscape in the general population. With the caveat that this cross-section of samples selects solely from adult individuals >18 years of age interacting with healthcare in primary or secondary settings, demographic data associated with these samples revealed that such an approach can offer a valuable insight into the diversity of antigenic exposures in the community (Table 1). In this study, 997 samples were derived from 959 unique patients. 81.5% of participants had received ≥1 dose of COVID-19 vaccine since 2020, while 69.4% had received ≥3 doses. The remaining 18.5% had no history of COVID-19 vaccination. Within the study group, annual COVID-19 vaccine coverage was 79.8% in 2021 (peak vaccine roll-out), falling to 56.9% in 2022, 41.2% in 2023, and 16.9% to date in 2024 (Table 1).

**Table 1:** Demographic details of the sample population.

Demographic data associated with the samples analysed in this study were obtained from the NHSGGC Safe Haven. All data were anonymized by the NHSGGC Biorepository. Vaccination coverage was calculated as detailed in the supplementary methods. NR = not reported, N/A = not applicable.

**Fig. 1:**
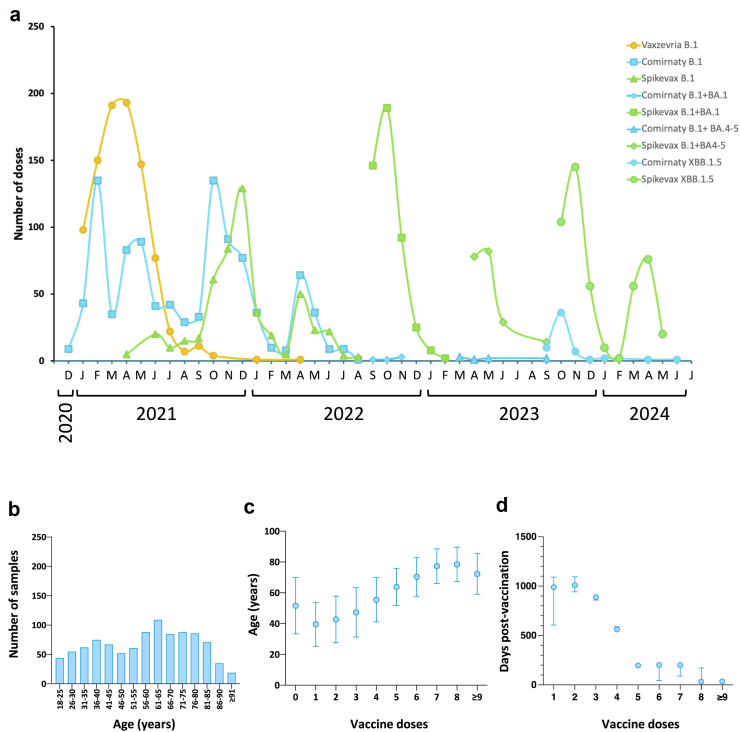
**Vaccine usage in the study population.** (a) COVID-19 vaccine types administered in each month were collated for the study population (n = 959 unique samples). Vaccines administered mirror the UK-wide rollout of each iteration, from monovalent B.1-derived vaccines through bi-valent B.1 + BA.1 to monovalent XBB.1.5-based vaccines. (b) Number of samples by patient age (years). (c) Age of patients having received each number of vaccine doses (mean ± SD). (d) Days since last vaccine administered for patients having received each number of vaccine doses (median (IQR)).

Analysis of the vaccine types administered reflected COVID-19 vaccine roll-out in the UK (Fig. 1a, Table 1), from the first introduction of Wuhan-Hu-1-derived Comirnaty (BNT162b2, Pfizer-BioNTech) and Vaxzevria (ChAdOx1, AstraZeneca), through to XBB.1.5 Spikevax (Moderna). “At-risk” individuals often received two doses of each vaccine iteration, hence, over the four-year period, many had received ≥7 to 8 doses of vaccine. Samples were collected from ages 19 to 98 (Fig. 1b), the mean age of the cohort increasing with number of doses (Fig. 1c), rising from a mean age of ∼40 for those receiving 1 or 2 doses, to 77–78 for those receiving 7 or 8 doses (Table 1, Fig. 1c), reflecting the targeting of elderly patients in booster vaccination campaigns.

To analyse the effect of multiple vaccine doses on the development and maintenance of the humoural response, samples were assigned into groups based on the number of vaccine doses received, and a subset of samples (n = 11–13) from each group were analysed for antibody responses to SARS-CoV-2. Where numbers permitted, samples were selected from patients who had received an XBB.1.5-based vaccine as their most recent vaccine dose, the rationale being that this would enable an assessment the impact of total number of doses administered and the effect of priming with non-XBB.1.5-based vaccines, upon the nature of the antibody response elicited. Hence, all samples in the 6, 7 and 8 dose groups had received 2 doses of XBB.1.5 vaccine during August 2023–March 2024 (Fig. 1a). Those receiving 4 or 5 doses had received 1 dose of XBB.1.5 vaccine, while those receiving ≤3 doses, had not received an XBB.1.5-derived vaccine. Those receiving ≤3 doses of vaccine had largely not been vaccinated for over 2 years, having received a primary course in 2021 and booster in 2022 (mean days since vaccination 855, Table 1, Fig. 1d), while those receiving ≥8 doses were vaccinated approximately 2 months prior to sample collection. These data underline the complex immunological landscape that now exists in the general population, where individuals differ in regard to number of doses, the type(s) of vaccines received, and time since last vaccination.

**Fig. 2:**
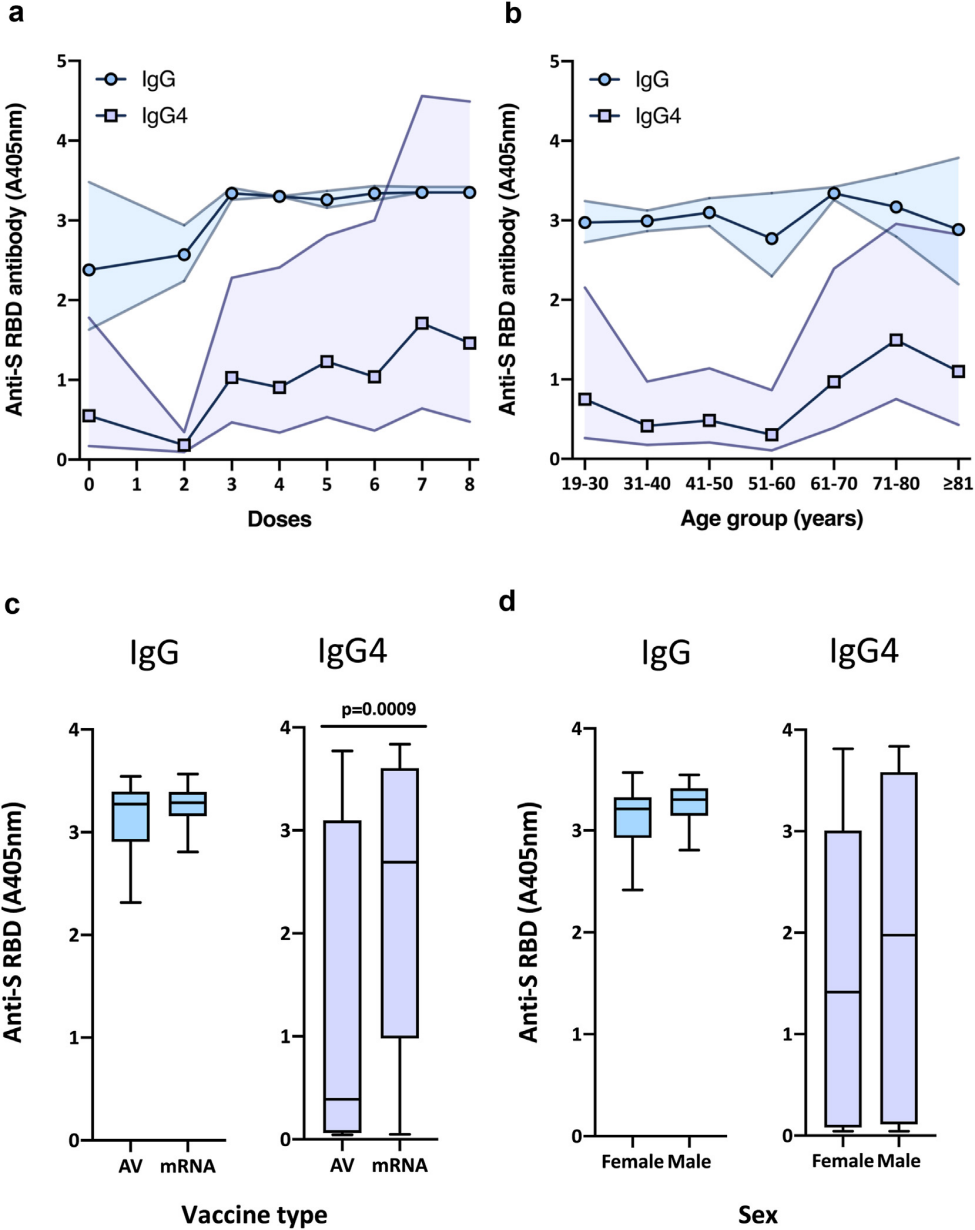
**Antibody response to SARS-CoV-2 RBD.** Levels of total IgG encompassing all sub-classes (IgG) or the IgG4 sub-class alone (IgG4) were measured by ELISA in duplicate. Responses (absorbance, 405 nm) are grouped by (a) number of vaccine doses received and (b) age groups of the patients (n = 108, geometric mean with 95% CI). All samples tested were then grouped on the basis of (c) the initial vaccine course received, either two doses of adenoviral-vectored vaccine (AV) (n = 51) or two doses of mRNA-based vaccine (mRNA) (n = 45), or (d) sex of the patient, whether female (n = 64) or male (n = 44). Boxplots represent median absorbance 405 nm with Tukey whiskers based on 25th percentile minus 1.5 × IQR and 75th percentile plus 1.5 × IQR. (c) IgG–AZ vs mRNA, not significant (p = 0.57), IgG4–AZ vs mRNA significant (p = 0.0009), (d) IgG–female vs male, not significant (p = 0.12), IgG4–female vs male, not significant (p = 0.25).

The level of SARS-CoV-2-specific immunoglobulins in each sample was measured by enzyme-linked immunosorbent assay (ELISA), measuring antibody binding to immobilised receptor-binding domain (RBD) of the viral spike protein (S). As previous studies have indicated that there may be substantial differences in the isotype of the immunoglobulins elicited post-vaccination with mRNA-based and non-mRNA-based vaccines,^23^^,^^25^^,^^26^ levels of total IgG, and IgG4 specifically were measured in parallel (Fig. 2). 99.3% of samples tested were positive for IgG antibodies against the SARS-CoV-2 RBD antigen. Total RBD-specific IgG levels were lower in samples from those receiving no vaccine, or 2 doses of vaccine. Samples from those receiving ≥3 doses had similar levels of total IgG (Fig. 2a). Analysis of IgG4 levels in the samples revealed a dose-dependent increase in RBD reactivity (Fig. 2a), consistent with the predominant use of mRNA-based COVID-19 vaccines in recent years. A similar pattern of increasing IgG4 levels was noted when the samples were stratified by age (Fig. 2b); IgG levels were similar across all age groups while IgG4 levels increased markedly ≥61 years, the groups that had received ≥5 doses of vaccine (≥3 doses of mRNA-based vaccine). Given the reported link between repeated doses of mRNA vaccine and elevated levels of SARS-CoV-2-specific IgG4,^25^^,^^26^ the samples were grouped solely on the basis of the first two doses of vaccine received, asking whether the primary course was adenoviral vectored (AV) or mRNA-based (mRNA). While anti-RBD IgG levels were similar in those having received a primary course of adenoviral vectored or mRNA-based vaccine (not significant, p = 0.57, Mann–Whitney U test; Fig. 2c), IgG4 levels were significantly higher (p = 0.0009, Fig. 2c) in samples from those having received a primary course of an mRNA-based vaccine, even although the samples were collected in May 2024, and the primary course had generally been administered in 2021. Finally, samples were stratified by sex (male or female). IgG and IgG4 levels were not significantly different between samples from male and female patients (IgG–female vs male, p = 0.12, IgG4–female vs male, p = 0.25; Fig. 2d).

**Fig. 3:**
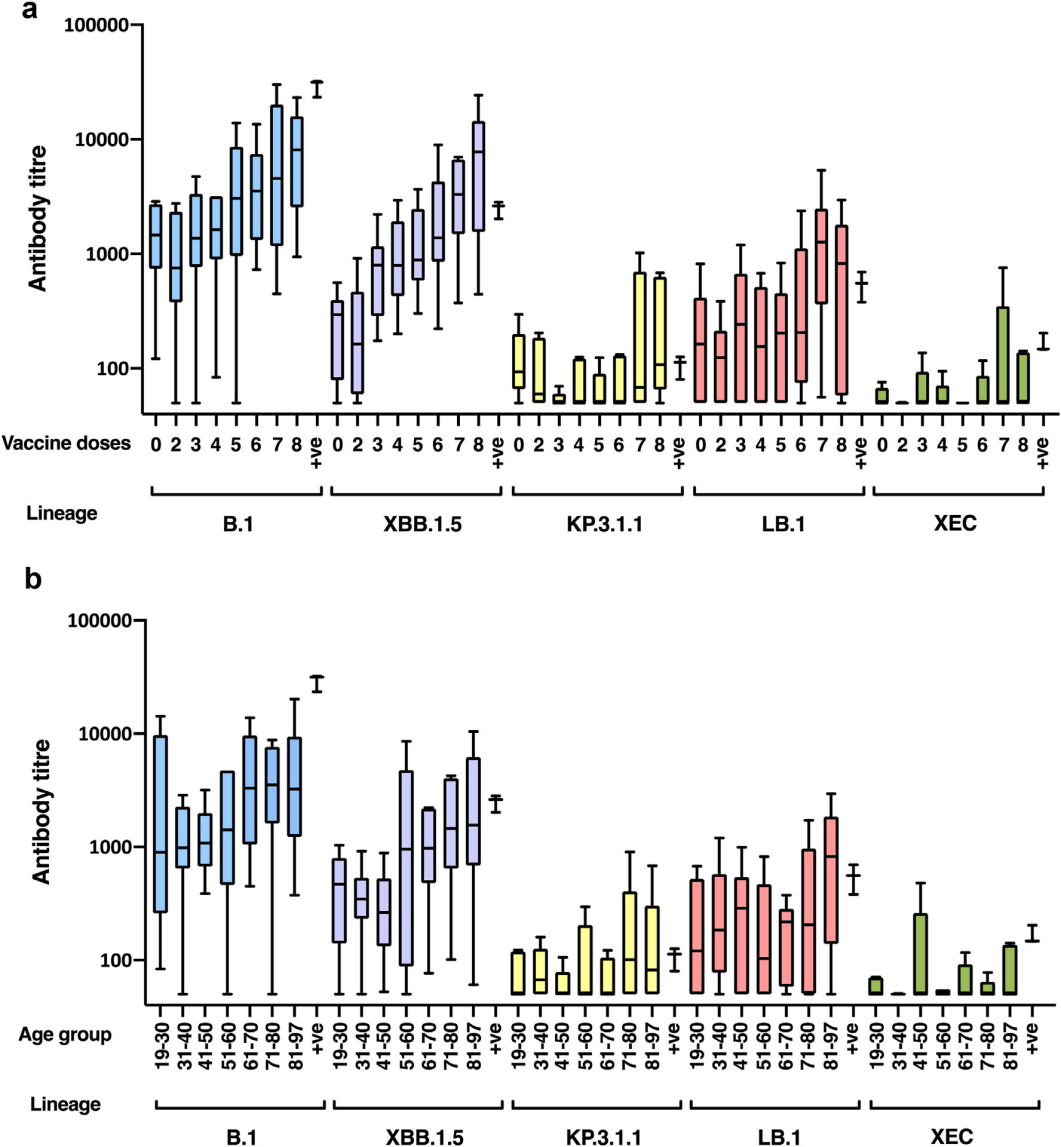
**Neutralising antibody response to SARS-CoV-2 variants.** (a) Neutralising activity against the vaccine antigens B.1 and XBB.1.5, and recent variants KP.3.1.1, LB.1 and XEC, were measured using HIV(SARS-CoV-2) pseudotypes bearing the respective spike proteins in triplicate. Samples (n = 108) were grouped based on number of vaccine doses received. +ve = purified IgG (KIOVIG). (b) Neutralising activity against B.1, XBB.1.5, KP.3.1.1, LB.1 and XEC stratified by age of the vaccinated individual. Boxplots display median with Tukey whiskers (based on 25th percentile minus 1.5 × IQR and 75th percentile plus 1.5 × IQR).

ELISAs for anti-RBD specific IgG and IgG4 revealed an effect of both dose and age on the strength of the response. We next investigated whether this was reflected in the titre of functional, neutralising antibodies against recent variants. Levels of neutralising antibodies in patient sera have been shown previously to correlate strongly with protection from infection.^35^, ^36^, ^37^, ^38^ Virus neutralising antibody (nAb) titres for each of the samples were quantified against the prototype vaccine antigen (B.1), the most recent vaccine antigen (XBB.1.5) to the sampling window, two JN.1-derived variants that were circulating at the time of sampling (KP.3.1.1 and LB.1), and the most recent variant to expand in the UK (XEC) (Supplementary Fig. S1), a variant which emerged in June 2024, immediately after the study sera were collected (May 2024). There was a vaccine dose-dependent increase in nAb titres against B.1 (Fig. 3), from a geometric mean titre of 1529 (95% CI, 608–3846) in patients having received no vaccination (presumably immunity elicited solely from natural exposure) to a titre of 5610 (95% CI, 2773–11,349) in those having received 8 doses (Supplementary Table S1). This corresponds to a geometric mean ratio (GMR) of 3.7 (95% CI, 1.2–11.7), suggesting a substantial increase in neutralisation post vaccination. A similar pattern was observed against XBB.1.5, from a titre of 267 (95% CI, 111–644) in unvaccinated individuals to a titre of 4577 (95% CI, 1832–11,440) in those receiving 8 doses of vaccine—GMR of 17.1 (95% CI 4.8–61.0) (Supplementary Table S1). Titres elicited against XBB.1.5 were lower than those against B.1 in the groups having received no vaccine (GMR 5.7, 95% CI 2.0–16.2) or two doses of vaccine (GMR 5.4, 95% CI 2.4–12.0) (p = 0.2 and p = 0.093 respectively, Friedman test), consistent with previous findings demonstrating reduced cross-neutralisation of XBB.1.5 following exposure to B.1 derived antigens.^34^ Samples from individuals in the 6, 7 and 8 dose groups, all of whom had received two doses of XBB.1.5 vaccine, displayed high titres of neutralising activity against XBB.1.5 (mean titres of 1703 (95% CI, 861–3371), 3080 (95% CI, 1439–6592), and 4577 (95% CI, 1832–1140) respectively, Supplementary Table S1). Cross-neutralising activity extended to variant LB.1 in the multiply vaccinated group, mean titres of 1666 and 945 after 7 and 8 doses respectively (Supplementary Table S1). Cross-neutralisation of KP.3.1.1 was limited in all groups, reaching maxima of 145 (95% CI, 62.2–336) and 187 (95% CI, 83.8–418) in the 7 and 8 dose groups respectively. Compared with responses against the vaccine antigens B.1 and XBB.1.5, titres against KP.3.1 were significantly reduced (Supplementary Table S2), for B.1 this was irrespective of number of vaccine doses (no doses: GMR 12.8, 95% CI 4.3–38.2, p = 0.0006; 2 doses: GMR 8.9, 95% CI 4.0–19.6, p < 0.0001; 3 doses: GMR 21.3, 95% CI 9.9–46.2, p < 0.0001; 4 doses: GMR 22.5, 95% CI 7.2–70.3, p = 0.0002; 5 doses: GMR 29.2, 95% CI 10.2–83.8, p = 0.0002; 6 doses: GMR 38.8, 95% CI 17.0–88.5, p < 0.0001; 7 doses: GMR 29.0, 95% CI 8.0–105, p = 0.0021; 8 doses: GMR 30, 95% CI, 10.3–87.3, p = 0.0021, Friedman test), while for XBB.1.5, this was significant in those having received 3–8 doses (3 doses: GMR 11.3, 95% CI 6.2–20.7, p = 0.01; 4 doses: GMR 13.1, 95% CI 4.5–38.3, p = 0.015; 5 doses: GMR 15.7, 95% CI 6.9–35.3, p = 0.0065; 6 doses: GMR 19.0, 95% CI 8.1–44.9, p = 0.0032; 7 doses: GMR 21.2, 95% CI 6.8–66.1, p = 0.0095; 8 doses: GMR 24.5, 95% CI 7.2–82.8, p = 0.037, Friedman test). When variants KP3.1.1 and LB.1 emerged, KP.3.1 subsequently became the dominant global variant while LB.1 diminished (Supplementary Fig. S1), consistent with KP.3.1.1 having an enhanced ability to evade neutralisation,^6^ irrespective of vaccine history. More recently, a new JN.1-derived variant emerged, designated “XEC”. This variant appears to be a recombinant between viruses of lineages KS.1.1 and KP.3.3 (Supplementary Fig. S1, Supplementary Table S3). Antibody titres against XEC were extremely low, titres of 65.2 (95% CI, 42.2–101), 60.5 (95% CI, 48.2–75.8) and 71.1 (95% CI, 46–110) in those having received no vaccine, or 2 and 3 doses respectively (Supplementary Table S1). Maximum XEC cross-neutralisation was achieved in the group having received 7 doses, with a titre of 105 (95% CI, 47–233). Compared with responses against the vaccine antigen B.1, titres against XEC were significantly reduced (Supplementary Table S2), irrespective of number of vaccine doses (no doses: GMR 23.5, 95% CI 8.5–65.1, p < 0.0001; 2 doses: GMR 15.5, 95% CI 7.6–31.5, p < 0.0001; 3 doses: GMR 17.8, 95% CI 7.6–42.0, p < 0.0001; 4 doses: GMR 23.0, 95% CI 6.7–78.6, p = 0.0004; 5 doses: GMR 28.5, 95% CI 9.4–87.0, p = 0.0004; 6 doses: GMR 43.2, 95% CI 18.1–103, p < 0.0001; 7 doses: GMR 40.0, 95% CI 11.4–141, p = 0.0001; 8 doses: GMR 61.7, 95% CI, 23.9–160, p < 0.0001, Friedman test). Similarly, when compared with responses against the vaccine antigen XBB.1.5, titres against XEC were also significantly reduced (Supplementary Table S2), irrespective of number of vaccine doses (no doses: GMR 4.1, 95% CI 1.5–10.9, p = 0.0062; 2 doses: GMR 2.9, 95% CI 1.8–4.7, p = 0.0009; 3 doses: GMR 9.4, 4.6–19.2, 95% CI, p = 0.013; 4 doses: GMR 13.3, 95% CI 4.1–43.1, p = 0.024; 5 doses: GMR 15.3, 95% CI 6.3–37.2, p = 0.01; 6 doses: GMR 21.2, 95% CI 8.6–52.1, p = 0.0002; 7 doses: GMR 29.3, 95% CI 9.7–88.5, p = 0.0007; 8 doses: GMR 50.3, 95% CI 16.5–154, p = 0.0012, Friedman test).

A similar reduction in antibody titre with successive variants was observed when the data were stratified based on the age of the individual (Fig. 3B, Supplementary Table S4) in comparison with number of doses (Fig. 3a). Given that successive vaccination campaigns targeted the most vulnerable individuals, offering repeated vaccinations to the elderly, the number of doses received increased with age (Fig. 1c).

Antibody levels (by ELISA) and antibody titre (by neutralisation assay) increased with the age of the study participants, and the number of vaccine doses received. However, given that those having received the most doses would have been vaccinated more recently, the effect of waning on antibody titre should be considered. As age increased, the number of doses each participant received also increased (Spearman r = 0.76), and the time since vaccination decreased (r = −0.6), elderly participants having received boosters more recently than younger participants with fewer doses (Supplementary Table. S5). There was a strong negative correlation between “Days since last vaccination” and “Doses” (r = −0.75), in that the time elapsed since individuals with fewer doses had received their last dose tended to be greater.

To examine the relationship between age, dose and a waning response we constructed a generalized additive model (GAM). The GAM was used to determine which variables affected antibody levels measured by ELISA most significantly, with a focus on the relationship between “Age”, “Doses” and “Days since last vaccination”. During model selection (see Supplementary Methods) we also found that the following terms influenced the antibody response: vaccine type; variant tested against (neutralisation data); sex (neutralisation data). In comparison with those elicited by the AstraZeneca adenoviral vaccine, IgG levels were found to be significantly higher in those vaccinated with the Pfizer mRNA vaccine (p = 0.0037, Wald test) (Supplementary Table S6). Further, the analysis suggested that for IgG, as “Days since last vaccination” increased, IgG levels decreased (p < 0.0001, Chi-sq test), confirming that total antibody levels were waning over time (Supplementary Table S7). The analysis also suggested that there was a complex relationship between IgG levels and both “Age, Doses” and “Doses, Days since last vaccination” (Supplementary Fig. S3). At fewer doses, the response was determined by “Days since last vaccination”, however as the number of doses increased, the days elapsed since vaccination decreased, therefore the response was less dependent on “Day since last vaccination” (Supplementary Fig. S3a). However, when looking at the interaction between “Age, Doses”, without “Days since last vaccination” (Supplementary Fig. S3b), then those participants having received fewer doses had higher IgG responses. Accordingly, we can infer that the higher responses in those with more doses, is due primarily to fewer days since their last vaccination, and that “Days since last vaccination” is the dominant force on IgG levels.

As with total IgG, individuals who received the Pfizer mRNA-based vaccine as a first dose displayed higher levels of IgG4 (p = 0.0066, Wald test) than those having received the AstraZeneca vaccine (Supplementary Table S8). In comparison, individuals having received ≥1 dose of XBB.1.5-derived vaccine generally had lower IgG4 levels than those having received no doses (p = 0.0031, Wald test), underlining the complex nature of the immunological landscape that now exists. It is likely that the nature of antigenic exposures differs between those who received XBB.1.5 boosters and those who did not. Those who received an XBB.1.5 booster will have received a novel antigen delivered by mRNA vaccine, while those who did not receive and XBB.1.5 vaccine, will be younger, have received fewer doses *per se*, a greater time will have elapsed since their last vaccination, and their current responses will have been augmented through natural exposure (infection). IgG4 levels decreased (p < 0.0001, Chi-sq test) as “Days since last vaccination” increased (Supplementary Table S9), consistent with IgG4 levels being the result of recent exposure to a potent immunogen.

Examining neutralising responses in the GAM confirmed that responses against all variants were significantly lower than those against the prototypic lineage B.1 antigen in the first vaccines (XBB.1.5, KP.3.1.1, LB.1 and XEC–p < 0.0001, (Wald test), Supplementary Table S10). For younger participants, those with fewer doses had higher responses. As “Age” and “Doses” increased, this effect diminished, however, the neutralising responses increased once again in the oldest people with the most doses (Supplementary Table S11, Supplementary Fig. S4). At fewer doses, there was a broad range of “Days since last vaccination”, those with fewer days since last vaccination had higher responses. As number of doses increased, the days since last vaccination decreased accordingly and the neutralising response increased.

## Discussion

During the COVID-19 pandemic, the Office for National Statistics (ONS) in the United Kingdom collated data that described the number of individuals testing positive for SARS-CoV-2 nucleic acid, and those with SARS-CoV-2-specific antibodies in their blood. The Coronavirus (COVID-19) Infection Survey was discontinued in March 2023, noting that 77.7%, 79.5%, 74.5% and 79.8% of adults aged 16 years or over from England, Wales, Northern Ireland and Scotland respectively had antibodies against SARS-CoV-2 ≥800 ng/ml in the tests used at the time.^39^ The end of routine serological surveillance studies post-COVID-19 pandemic has meant that data on the level of immunity present in the general population are scarce. Importantly, as SARS-CoV-2 lineages continue to evolve, few studies are now tracking the level of immune evasion by emerging variants, and hence likely vaccine effectiveness. Those that have continued, tend to be focussing on key target populations, for example the elderly or healthcare workers. However, little is known about the level of immunity in the general population, the impact of vaccination and natural infection on immunity and the level of immune evasion by current variants. In this study, we show that analysing samples from the biorepository of a major health board provides a simple means of monitoring the immunological landscape of a representative portion of the population. Our results provide an insight into the immunological impacts of the many, diverse COVID-19 vaccine histories that now exist within the community. While SARS-CoV-2 infection histories are now poorly documented, this analysis revealed that it was possible to discriminate a clear dose-dependent increase in levels of neutralising antibody with vaccine dose history. The data generated in this study suggest that the replicative advantage of KP.3.1.1 and subsequently, XEC, stems largely from the high level of evasion of the neutralising response. Significant levels of neutralisation against KP.3.1.1 and XEC were only present in those individuals having received 7 or 8 doses in total, culminating in 2 doses of the most recent XBB.1.5 vaccine. Importantly, in those having received 6 doses in total, the final 2 doses of the XBB.1.5 vaccine were still insufficient to induce significant cross-neutralisation of both KP.3.1.1 and XEC. As the group receiving ≥7 doses of vaccine represented approximately 19% of the samples collected, this suggests that the majority of the general population (≥80%) likely have insufficient cross-neutralising activity to control infection with current variants. While the majority of such individuals may well display mild respiratory symptoms akin to that of infection with seasonal coronaviruses, relying on pre-existing cross-reactive immune responses to prevent severe clinical disease, “at-risk” individuals may struggle to control current variants unless they remain fully vaccinated. Current guidelines in which older individuals (≥65 years) and those at risk of impaired immune function are offered seasonal COVID-19 boosters with the most recent iterations of COVID-19 vaccines (currently derived from the JN.1 lineage) would seem prudent based on the cross-neutralising activity detected in samples from those vaccinated with ≥7 doses. The generalized additive model indicated that “Days since last vaccination” was a significant term, with the antibody response waning as the number of days increased. The higher antibody responses seen in older individuals having received more doses, was significantly influenced by them having their booster vaccinations more recently. Accordingly, these data support the continued, annual vaccination of those most at risk from disease.

Consistent with recent reports,^23^, ^24^, ^25^, ^26^ we found that repeated vaccination with mRNA-based COVID-19 vaccines led to a marked degree of class-switching to IgG4. As repeated mRNA booster vaccinations have been restricted largely to the elderly and the immunocompromised, there is now a complex immunological landscape in which there is an age-related increase in IgG4 levels, an immunoglobulin isotype with substantially reduced Fc-mediated functions such as Fc-receptor binding and complement fixation. It remains unclear whether this may be beneficial (regulating the extent of the immune response), or detrimental (preventing Fc-mediated activities such as antibody-dependent cell killing or phagocytosis). Indeed, it has been suggested that a more considered approach to vaccination with mRNA-based vaccines may be appropriate, for example spacing out booster vaccines more widely or using heterologous boosts with non-mRNA-based vaccines.^24^

In comparison with JN.1 (the variant that evolved and spread globally from the founder BA.2.86), it is striking how few amino acid substitutions in the spike proteins of KP.3.1.1 and XEC are required to render the virus highly immune evasive. The KP.3.1.1 spike is effectively that of JN.1 with S31-, F456L, Q493E, V1104L, while the XEC spike is JN.1 with T22N, F59S, F456L, Q493E and V1104L (Supplementary Figs. S1 and S2). Whether the new JN.1-derived vaccines introduced in Autumn 2024 will be sufficient to extend immunity in at-risk populations to control the most recent variants remains to be seen.

In this study, we examined whether analysing a random selection of samples from the Biorepository serving a major hospital in Glasgow could enhance our understanding of the level of population immunity to SARS-CoV-2. While valuable insights were provided into the level of immune evasion by current variants, and the influence of age, number of doses of vaccine received, and the importance of waning responses, such studies have limitations. The samples used in this study were collected from a healthcare-seeking population residing largely in the South of Glasgow, as such the study population may have biases relative to the general population. The majority of SST samples submitted to the Biochemistry service request urea and electrolytes (U&E) and hence are not sourced from patients with a narrow range of conditions. Rather, a “U&E” with full blood count (FBC) would be a standard request for a broad range of patients, from a generally unwell member of the public attending a general practitioner, to a hospital patient in intensive care. How this healthcare-seeking population compares immunologically with the general population as a whole should be the focus of future studies, to ascertain whether such samples represent a cost-effective insight into the current level of immunity to viral pathogens.

The immunological landscape of the general population is now complex, with most people displaying “hybrid immunity”, elicited through a combination of vaccinations and infections. Recent studies have suggested that the way natural infection is influencing immunity has shifted since the emergence of the Omicron variant. Prior to Omicron, natural infection elicited potent, long-lasting immunity. Post-Omicron, infections stimulate short-lived immunity, that wanes rapidly.^40^ If public health agencies are to make informed decisions about strategies for COVID-19 vaccination going forward, a program of annual or biannual sampling from the general population may be prudent to advise vaccine antigen selection and timing going forward. Random sampling from a selection of the biorepositories of major general hospitals across the UK may represent a simple and cost-effective approach to monitoring the immunological landscape.

## Contributors

BJW, MJH and MM conceptualised the study and methodology. SR coordinated sample reception, storage and retrieval prior to analyses. PRM coordinated ethical approval and sample acquisition from the biorepository. JN, DB, IS and BW coordinated design and synthesis of the variant spike expression constructs. NL, BJW and SS optimised and performed the laboratory analyses and collected the data. MM and BJW accessed, verified and analysed the underlying data and wrote the original and revised copies of the manuscript. ECT, MJH, PRM, JN, MM and BJW reviewed and edited the manuscript with input from all authors. All authors had full access to the data in the study, read and approved the final version of the manuscript, and had final responsibility for the decision to submit for publication.

## Data sharing statement

Deidentified metadata from this study, and constructs for viral pseudotyping (subject to availability) are available from the corresponding author upon reasonable request.

## Declaration of interests

PRM declares funding from UKRI (MC_UU_0034/2), BBSRC (BB/V002821/1, BB/V004697/1), HBLB (Prg797, Prg816) and MSD (MISP 101910). ECT declares funding from MRC World Class Labs (MC_PC_ MR/Y002814/1), Novavax, Astra Zeneca, University of Oxford, University of Southampton, Moderna (Vaccine trial research (COVID-19 vaccines), EEID - BBSRC, (CCHFV research), UKRI (OHCN computational research), NIHR (NIHR research professorship). All other authors declare no competing interests.

## Appendix A Supplementary data

Supplementary methods

Captions for Supplementary Tables and Figures
