# Supplementary material for: Estimating population immunity to SARS-CoV-2 by random sampling from primary and secondary healthcare in Scotland, May 2024
Source: eBioMedicine. 2025 May 16;116:105760. doi: 10.1016/j.ebiom.2025.105760 (PMC12146547; doi:10.1016/j.ebiom.2025.105760)
Supplement: Supplementary Table S1 [file mmc1.docx]

**Supplementary Table S1: Neutralising antibody titres stratified by dose**

**Supplementary Table S1: Neutralising antibody titres stratified by dose (continued)**
