# Supplementary material for: Estimating population immunity to SARS-CoV-2 by random sampling from primary and secondary healthcare in Scotland, May 2024
Source: eBioMedicine. 2025 May 16;116:105760. doi: 10.1016/j.ebiom.2025.105760 (PMC12146547; doi:10.1016/j.ebiom.2025.105760)
Supplement: Supplementary Table S2 [file mmc2.docx]

**Supplementary Table S2. Relationship between number of vaccine doses and antibody titre, comparison between variants at each dose.**

**Supplementary Table S2. Effect of number of vaccine doses on antibody titre, comparison between variants at each dose (continued).**
