# Supplementary material for: Estimating population immunity to SARS-CoV-2 by random sampling from primary and secondary healthcare in Scotland, May 2024
Source: eBioMedicine. 2025 May 16;116:105760. doi: 10.1016/j.ebiom.2025.105760 (PMC12146547; doi:10.1016/j.ebiom.2025.105760)
Supplement: Supplementary Table S5 [file mmc5.docx]

**Table S5. Correlation between Age, Dose and Days since last vaccination.**

**CI = confidence interval**

| **Term** | **Correlation** | **95% CI** | **P-value** |
| --- | --- | --- | --- |
| **Age vs Dose** | 0.76 | 0.66 – 0.83 | <0.0001 |
| **Age vs Days since last vaccination** | -0.60 | -0.71 – -0.48 | <0.0001 |
| **Doses vs Days since last vaccination** | -0.75 | -0.84 – -0.64 | <0.0001 |
