# Supplementary material for: Estimating population immunity to SARS-CoV-2 by random sampling from primary and secondary healthcare in Scotland, May 2024
Source: eBioMedicine. 2025 May 16;116:105760. doi: 10.1016/j.ebiom.2025.105760 (PMC12146547; doi:10.1016/j.ebiom.2025.105760)
Supplement: Supplementary Table S6 [file mmc6.docx]

**Table S6. Relationship between IgG levels and vaccine type.**

| **Variable** | **Coefficient estimate** | **95% CI** | **P-value** | **Interpretation** |
| --- | --- | --- | --- | --- |
| **Intercept** | 3.1 | 3.06, 3.14 | <0.0001 | Baseline IgG if predictors at reference levels |
| **Vaccine type**  **Pfizer** | 0.11 | 0.034, 0.19 | 0.0034 | Pfizer significantly increases IgG compared to AZ |

CI = confidence interval. Derived from a generalised additive model (GAM).
