# Supplementary material for: Estimating population immunity to SARS-CoV-2 by random sampling from primary and secondary healthcare in Scotland, May 2024
Source: eBioMedicine. 2025 May 16;116:105760. doi: 10.1016/j.ebiom.2025.105760 (PMC12146547; doi:10.1016/j.ebiom.2025.105760)
Supplement: Supplementary Table S7 [file mmc7.docx]

**Table S7. Relationship between IgG levels and Age, Dose and Days since last vaccination.**

| **Smooth term** | **Estimated degrees of freedom** | **Reference degrees of freedom** | **Chi.sq** | **P-value** | **Interpretation** |
| --- | --- | --- | --- | --- | --- |
| **Days since last vaccination** | 1.0 | 1 | 28.4 | <0.0001 | As Days since last vaccination increases, IgG levels decrease |
| **Age, Doses** | 3.0 | 3 | 15.8 | 0.0012 | Complex relationship between IgG and Age, Doses |
| **Doses, Days since last vaccination** | 10.7 | 23 | 100 | <0.0001 | Complex relationship between IgG and Doses, Days since last vaccination |

Derived from a generalised additive model (GAM).
