# Supplementary material for: Estimating population immunity to SARS-CoV-2 by random sampling from primary and secondary healthcare in Scotland, May 2024
Source: eBioMedicine. 2025 May 16;116:105760. doi: 10.1016/j.ebiom.2025.105760 (PMC12146547; doi:10.1016/j.ebiom.2025.105760)
Supplement: Supplementary Table S8 [file mmc8.docx]

**Table S8. Relationship between IgG4 levels and vaccine type.**

| **Variable** | **Coefficient estimate** | **95% CI** | **P-value** | **Interpretation** |
| --- | --- | --- | --- | --- |
| **Intercept** | 4.4 | 3.2, 5.6 | <0.0001 | Baseline IgG4 |
| **Vaccine type**  **Pfizer** | 0.51 | 0.14, 0.88 | 0.0066 | Pfizer significantly increases IgG4 |
| **Doses**  **XBB.1.5 ≥1** | -2.2 | -3.7, -0.73 | 0.0031 | Individuals with ≥1 XBB.1.5 booster have lower IgG4 than those who received no booster |

CI = confidence interval. Derived from a generalised additive model (GAM).
