# Supplementary material for: Estimating population immunity to SARS-CoV-2 by random sampling from primary and secondary healthcare in Scotland, May 2024
Source: eBioMedicine. 2025 May 16;116:105760. doi: 10.1016/j.ebiom.2025.105760 (PMC12146547; doi:10.1016/j.ebiom.2025.105760)
Supplement: Supplementary Table S9 [file mmc9.docx]

**Table S9. Relationship between IgG4 levels and Days since last vaccination.**

| **Smooth term** | **Estimated degrees of freedom** | **Reference degrees of freedom** | **Chi.sq** | **P-value** | **Interpretation** |
| --- | --- | --- | --- | --- | --- |
| **Days since last vaccination** | 1.00 | 1 | 17.2 | <0.0001 | As Days since last vaccination increases, IgG4 levels generally decrease |

Derived from a generalised additive model (GAM).
