# Supplementary material for: Estimating population immunity to SARS-CoV-2 by random sampling from primary and secondary healthcare in Scotland, May 2024
Source: eBioMedicine. 2025 May 16;116:105760. doi: 10.1016/j.ebiom.2025.105760 (PMC12146547; doi:10.1016/j.ebiom.2025.105760)
Supplement: Supplementary Table S10 [file mmc10.docx]

**Table S10. Relationship of neutralising antibody titre with viral variant and sex.**

| **Variable** | **Coefficient estimate** | **95% CI** | **P-value** | **Interpretation** |
| --- | --- | --- | --- | --- |
| **Intercept** | 6.8 | 6.5, 7.1 | <0.0001 | Baseline nAb titre |
| **XBB.1.5** | -0.74 | -1.1, -0.41 | <0.0001 | Lower titre than for B.1 |
| **KP.3.1.1** | -2.7 | -3.1, -2.3 | <0.0001 | Lower titre than for B.1 |
| **LB.1** | -1.8 | -2.2, -1.4 | <0.0001 | Lower titre than for B.1 |
| **XEC** | -2.7 | -3.1, - 2.3 | <0.0001 | Lower titre than for B.1 |
| **(Sex)Male** | 0.38 | 0.11, 0.65 | 0.0055 | Males have higher nAb titres than females |

CI = confidence interval. Derived from a generalised additive model (GAM).
