# Supplementary material for: Estimating population immunity to SARS-CoV-2 by random sampling from primary and secondary healthcare in Scotland, May 2024
Source: eBioMedicine. 2025 May 16;116:105760. doi: 10.1016/j.ebiom.2025.105760 (PMC12146547; doi:10.1016/j.ebiom.2025.105760)
Supplement: Supplementary Table S11 [file mmc11.docx]

**Table S11. Relationship between neutralising antibody titre and Age, Dose and Days since last vaccination.**

| **Smooth term** | **Estimated degrees of freedom** | **Reference degrees of freedom** | **Chi.sq** | **P-value** | **Interpretation** |
| --- | --- | --- | --- | --- | --- |
| **Age, Doses** | 8.9 | 11.5 | 37.3 | 0.00015 | Complex relationships between Age and Doses with titre |
| **Doses, Days since last vaccination** | 4.0 | 27.0 | 12.9 | 0.00099 | Complex relationships between Doses and Days since last-vacc with titre |

Derived from a generalised additive model (GAM).
