# Supplementary figures and images for: Estimating population immunity to SARS-CoV-2 by random sampling from primary and secondary healthcare in Scotland, May 2024
Source: eBioMedicine. 2025 May 16;116:105760. doi: 10.1016/j.ebiom.2025.105760 (PMC12146547; doi:10.1016/j.ebiom.2025.105760)

### Supplementary Figure S1

A

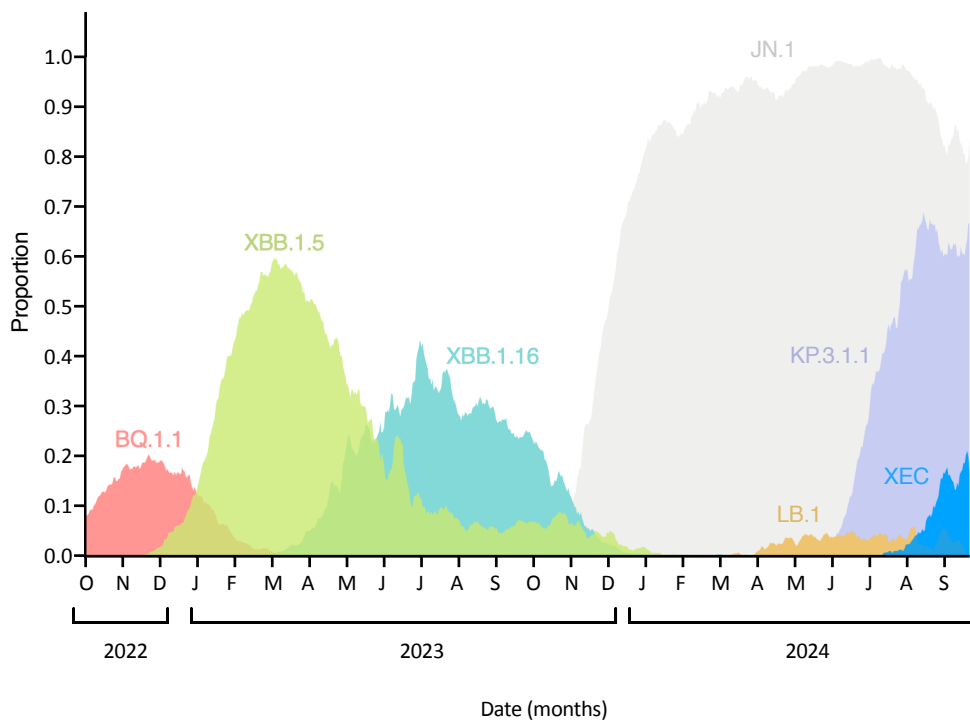

B

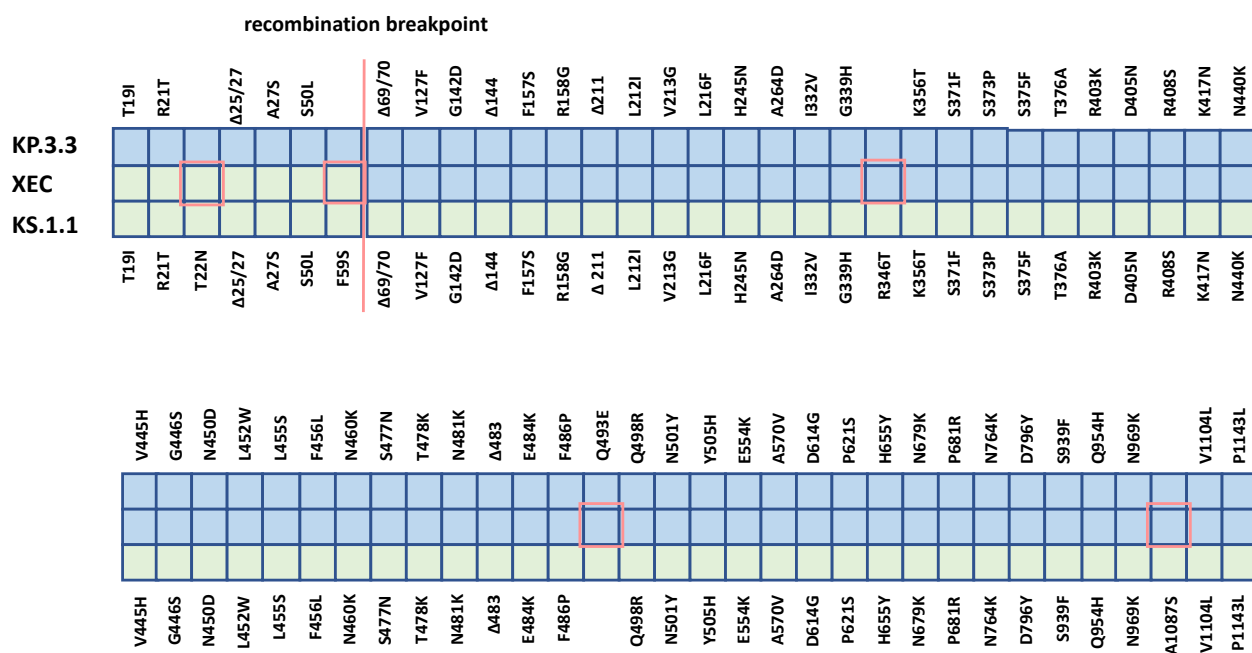

Supplement: Supplementary Figure S1 [file mmc12.pdf]

**Supplementary Figure S2. Location of amino acid substitutions in the XEC spike relative to JN.1**

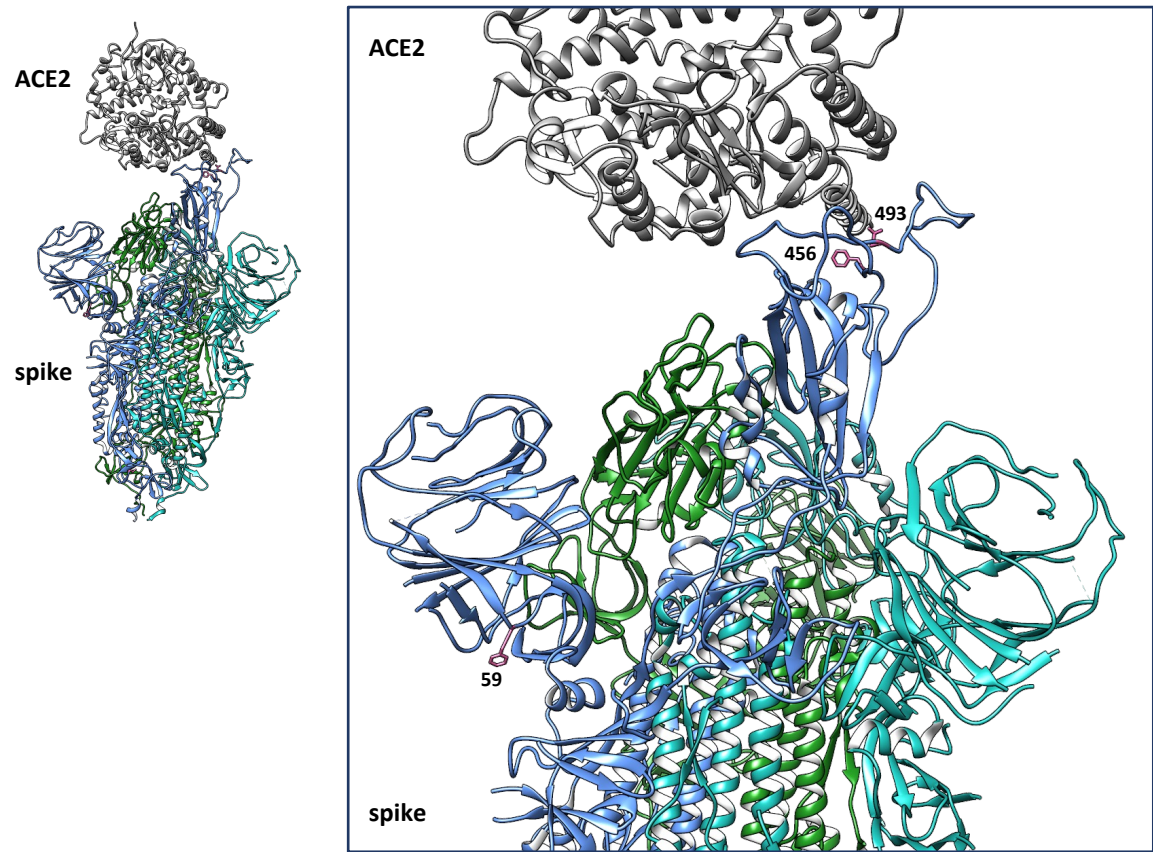

Supplement: Supplementary Figure S2 [file mmc13.pdf]
