# Supplementary material for: Estimating population immunity to SARS-CoV-2 by random sampling from primary and secondary healthcare in Scotland, May 2024
Source: eBioMedicine. 2025 May 16;116:105760. doi: 10.1016/j.ebiom.2025.105760 (PMC12146547; doi:10.1016/j.ebiom.2025.105760)

Supplementary Figure S4. Relationship between neutralising antibody titre and Age, Doses, and Days since last vaccination.

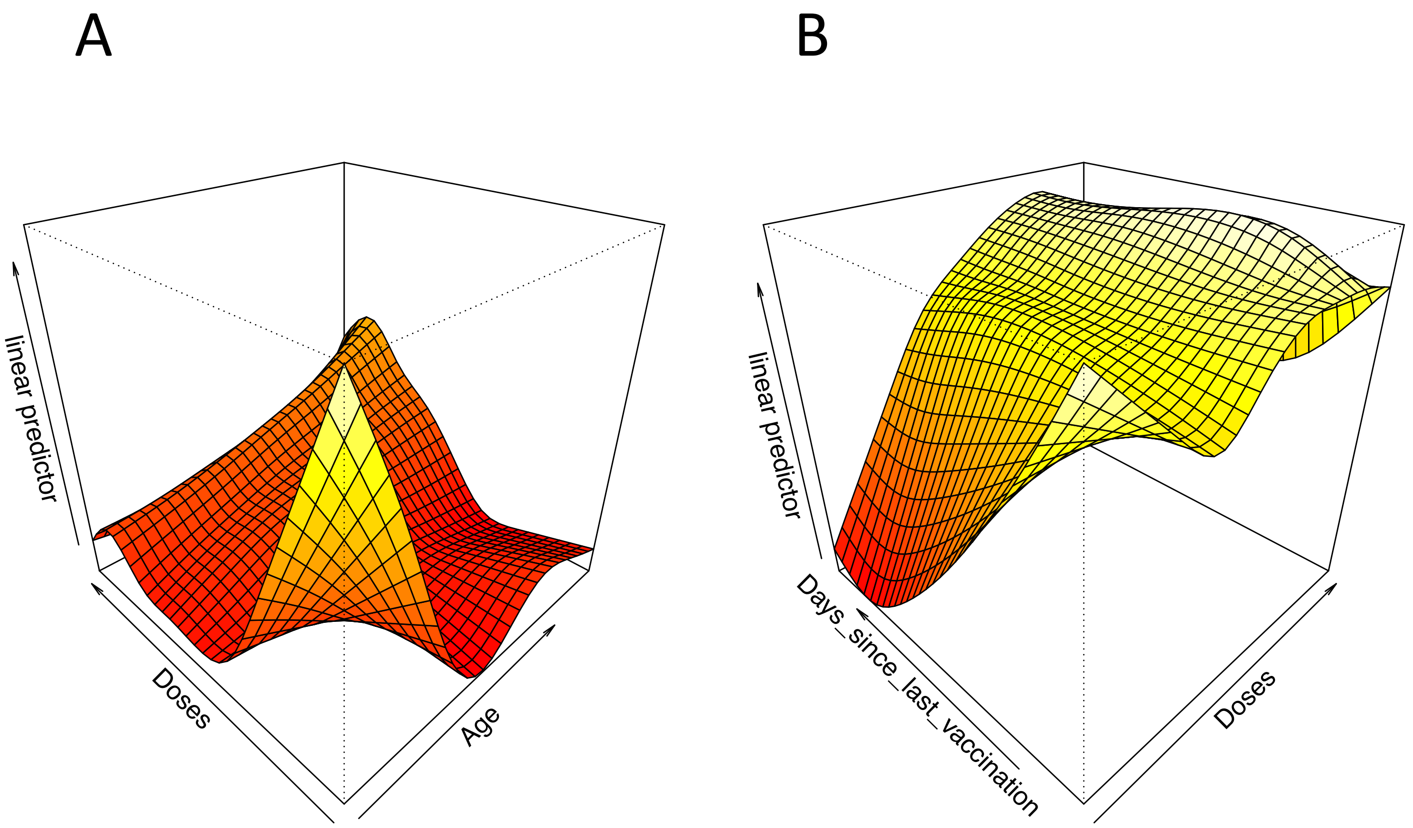

Supplement: Supplementary Figure S4 [file mmc15.pdf]
