# Supplementary material for: Estimating population immunity to SARS-CoV-2 by random sampling from primary and secondary healthcare in Scotland, May 2024
Source: eBioMedicine. 2025 May 16;116:105760. doi: 10.1016/j.ebiom.2025.105760 (PMC12146547; doi:10.1016/j.ebiom.2025.105760)
Supplement: Supplementary methods [file mmc16.docx]

**Supplementary methods**

**SARS-CoV-2 spike protein expression constructs**

Expression vectors encoding codon-optimized genes for the B.1 (Wuhan-Hu-1; D614G), XBB.1.5, KP.3.1.1, LB.1 and XEC were synthesised by Genscript. The constructs bore the following mutations relative to the ancestral Wuhan-Hu-1 sequence (GenBank: MN908947): **B.1** - D614G; **XBB.1.5** - T19I, L24-, P25-, P26-, A27S, V83A, G142D, Y144-, H146Q, Q183E, V213E, G252V, G339H, R346T, L368I, S371F, S373P, S375F, T376A, D405N, R408S, K417N, N440K , V445P, G446S, N460K, S477N, T478K, E484A, F486P, F490S, Q498R, N501Y, Y505H, D614G, H655Y, N679K, P681H, N764K, D796Y, Q954H, N969K; **KP.3.1.1 –** ins16MPLF, T19I, R21T, L24-, P25-, P26-, A27S, S31-, S50L, H69-, V70-, V127F, G142D, Y144-, F157S, R158G, N211-, L212I, V213G, L216F, H245N, A264D, I332V, G339H, K356T, S371F, S373P, S375F, T376A, R403K, D405N, R408S, K417N , N440K, V445H, G446S, N450D, L452W, L455S, F456L, N460K, S477N, T478K, N481K, V483-, E484K, F486P, Q493E, Q498R, N501Y, Y505H, E554K, A570V, D614G, P621S, H655Y, N679K, P681R, N764K, D796Y, S939F, Q954H, N969K, V1104L, P1143L; **LB.1 –** ins16MPLF, T19I, R21T, L24-, P25-, P26-, A27S, S31-, S50L, H69-, V70-, V127F, G142D, Y144-, F157S, R158G, Q183H, N211-, L212I, V213G, L216F, H245N, A264D, I332V, G339H, R346T, K356T, S371F, S373P, S375F, T376A, R403K, D405N, R408S, K417N , N440K, V445H, G446S, N450D, L452W, L455S, F456L, N460K, S477N, T478K, N481K, V483-, E484K, F486P, Q498R, N501Y, Y505H, E554K, A570V, T572I, D614G, P621S, H655Y, N679K, P681R, N764K, D796Y, S939F, Q954H, N969K, P1143L; **XEC** ins16MPLF, T19I, R21T, T22N, L24-, P25-, P26-, A27S, S50L, F59S, H69-, V70-, V127F, G142D, Y144-, F157S, R158G, N211-, L212I, V213G, L216F, H245N, A264D, I332V, G339H, K356T, S371F, S373P, S375F, T376A, R403K, D405N, R408S, K417N , N440K, V445H, G446S, N450D, L452W, L455S, F456L, N460K, S477N, T478K, N481K, V483-, E484K, F486P, Q493E, Q498R, N501Y, Y505H, E554K, A570V, D614G, P621S, H655Y, N679K, P681R, N764K, D796Y, S939F, Q954H, N969K, V1104L, P1143L.

**Serum samples**

The NHSGGC Biorepository is a resource for clinical research based at the Queen Elizabeth University Hospital (QUEH), Glasgow. The Biorepository provides access to a wide range of human tissue samples including surplus materials from diagnostic and surgical procedures, sourcing fully consented surplus tissue and other for use in studies requiring human tissue. Each anonymised sample is associated with a limited set of clinical and demographic data held by the Biorepository, working in close collaboration with the [NHSGGC Safe Haven](https://www.nhsggc.scot/hospitals-services/services-a-to-z/west-of-scotland-safe-haven/). The Biorepository is accredited by the United Kingdom Accreditation Service (UKAS) to ISO 20387:2018 standards. For this study, random residual biochemistry serum samples (n=997) from primary (general practices) and secondary (hospitals) health care settings were collected by the NHSGGC Biorepository between the 14th of May 2024 and 30th of May 2024. Twice a week, bulk collections of serum separating tubes (SST) containing samples surplus to diagnostic requirements, were retrieved from the QUEH Biochemistry department. The samples came from any hospital departments, or general practices within the south of Glasgow, that routinely send their SST samples to the QEUH Biochemistry department. Samples were selected from the discarded samples at random, confirmed to be from donors ≥18 years and to contain a sample volume sufficient to provide a 0.5ml aliquot. Associated metadata included age, care type, date of sample collection, date of positive COVID-19 PCR results, date of COVID-19 vaccination, and vaccine manufacturer were retrieved from the NHSGGC Safe Haven.

The subset used for experimental analysis included samples from 108 individuals. These samples were from participants where XBB.1.5 was their last dose to account for the influence of this on an individual’s antibody response. Participants were grouped by the number of doses they had received and within this, we randomly selected 6 males and 6 females for each group. Post-analysis, during data checking, samples were found to have "last_vaccine" dates after the sampling date and hence were moved to the appropriate group with one dose less (hence group sizes are 11-13). For those with 2 doses, we included 24 participants to balance the vaccine types participants had received. The final number of participants per each dose group was: no vaccine – n=12; 2 doses – n=24; 3 doses – n=13; 4 doses – n=11; 5 doses – n=13; 6 doses – n=13; 7 doses – n=11; 8 doses – n=11.

**Statistical analyses**

**Calculation of neutralising antibody titre by interpolation.**  The linear interpolation method effectively performs a linear interpolation between two points. The luciferase activity measured in the no serum control wells are multiplied by 0.5 to estimate a 50% reduction in infectivity, this defines a value "X". The linear interpolation finds the relative position between two known values in the dilution series range (above and below the value of X). It then uses this relative position to interpolate between the corresponding values in the dilution series range. In practice, the formula used is:

=(AN2-@INDEX(AN6:AN13,AN3))/(@INDEX(AN6:AN13,AN3+1)-@INDEX(AN6:AN13,AN3))*(@INDEX(AM6:AM13,AN3+1)-@INDEX(AM6:AM13,AN3))+@INDEX(AM6:AM13,AN3)

Where AN2 is the value of X (no serum control * 0.5), and AN3 is derived by using the formula =MATCH(AN2,AN6:AN13), with AM6:AM13 being the sample dilutions, and AN6:AN13 being the luciferase counts for the respective sample dilutions. The formula returns an estimate for the antibody titre. This is repeated for three replicates to give the mean titre. Antibody titres for each group were compared by One way ANOVA (Graphpad Prism) using the Friedman test, comparing the mean rank of each column with every other column. Additionally, the Geometric Mean Ratio (GMR) for comparing titres of two groups was calculated by dividing the geometric means of the two groups, and its confidence interval (CI) is derived by log-transforming the means, calculating standard errors, and then exponentiating the results to the original scale.

**Estimating annual vaccine coverage.** For each calendar year, participants were divided into those who either received at least one dose of vaccine, or who were not vaccinated. Where individuals received more than one dose per calendar year, they were recorded as simply as "vaccinated". We used the term "vaccine coverage" to align with annually reported figures during the COVID-19 pandemic, where vaccine coverage represents the percentage of the total study population who were vaccinated at least once in that calendar year.

**Assessing the correlation between groups.** The correlation between "Age", "Doses" and "Days since last vaccination" was assessed using a Spearman’s test (RStudio corr(data)).

**Data analysis using a generalized additive model (GAM).** The relationship between antibody responses and "Age", number of vaccine doses ("Doses"), time elapsed since last vaccination was administered ("Days since last vaccination") was assessed in R using RStudio. Initial analysis using a linear regression model, displayed poor predictive power and indicated a non-linear relationship with complex interactions between the variables. In the GAM (performed using the package mgcv in RStudio), titres were log3 transformed as the serial dilution for the titrations were performed in 3 fold steps. As "Age", "Doses" and "Days since last vaccination" were highly correlated, and multicollinearity can bring uncertainty to the model, a penalisation adjustment was included (ridge penalty, introducing a term that controls overfitting due to collinearity). The “te” function was used to investigate the complex relationship between variables. The assumptions for the GAM, including the independence, normality (using Q-Q plots), and homogeneity of the residuals (using residual plots, specifically residual versus fitted values), were checked and met.

The GAM examined the following variables:

Age: s(Age, k = 5) +

Doses: s(Doses, k=5) +

Days_since_last_vaccination: s(Days_since_last_vaccination, k=4) +

Sex: factor(Sex) +

Vaccine_type: factor(X1st.course) +

Doses_XBB.1.5 factor(XBB_1_5_doses) +

Care_type: factor(CareType) +

Postcode: factor(PostcodePart) +

Doses: te(Age, Doses, k=5) +

Age, Days_since_last_vaccination: te(Age, Days_since_last_vaccination, k = 5) +

Doses, Days_since_last_vaccination: te(Doses, Days_since_last_vaccination, k = 5),

For analysis of neutralising antibody responses, the additional variables were:

XBB.1.5 titre: factor(Variant)XBB_1_5

KP.3.1.1 titre: factor(Variant)KP_3_1_1

LB.1 titre: factor(Variant)LB_1

XEC titre: factor(Variant)XEC

Sex: factor(Sex)Male

"s" (spline) - if outcome is a continuous numerical output, used for non-linear relationships; "factor" - if output is non numerical or has a couple inputs (not continuous); "te" - interaction terms, if there is a suspected relationship between these terms
