# Supplementary material for: Estimating population immunity to SARS-CoV-2 by random sampling from primary and secondary healthcare in Scotland, May 2024
Source: eBioMedicine. 2025 May 16;116:105760. doi: 10.1016/j.ebiom.2025.105760 (PMC12146547; doi:10.1016/j.ebiom.2025.105760)
Supplement: Captions for Supplementary Tables and Figures [file mmc17.docx]

**Legends for Supplementary Figures and Tables**

**Supplementary Figure S1**. (A) Proportion of viral variants in circulation from October 2022 to September 2024, spanning before and after the May 2024 study period. (B) Amino acid sequence variation in XEC and its ancestors KP.3.3 and KS.1.1 relative to ancestral B.1. Predicted recombination breakpoint is shown. Key differences are boxed in red. Data were downloaded from CoV-Spectrum.org. November 2024. Blue boxes mark KP.3.3 derived sequences, green boxes mark KS.1.1 derived sequences.

**Supplementary Figure S2**. **Location of amino acid substitutions in the XEC spike relative to JN.1.** XEC differs at F59S, F456L and Q493E (shown in pink), and F59S and V1104 (not shown). The substitutions F456L and Q493E are located at the predicted site of interaction between spike and ACE2. Trimeric spike monomers are shown in green, cyan and blue; ACE2 is shown in grey.

**Supplementary Figure S3**. **Graphical representation of the relationship between total IgG levels and Doses, Age, and Days since last vaccination.** A. 3D surface plot displaying the interactions between IgG, "Age" and "Doses". B. 3D surface plot displaying the interactions between IgG, "Doses" and "Days since last vaccination". The linear predictor represents the predicted IgG levels, incorporating the combined effects of "Age" "Doses" and "Days since last vaccination". Higher values correspond to greater predicted IgG levels.

**Supplementary Figure S4**. **Relationship between neutralising antibody titre and Age, Doses, and Days since last vaccination.** A. 3D surface plot displaying the interactions between neutralising antibody titre, Age and Doses. B. 3D surface plot displaying the interactions between IgG, "Doses" and "Days since last vaccination". The linear predictor represents the predicted log₃ neutralising antibody titre, incorporating the combined effects of "Age", "Doses" Days since last vaccination". Higher values correspond to greater predicted titres.

**Supplementary Table legends**

**Supplementary Table S1**: **Descriptive statistics for antibody titres against each of the variants examined in the study.** Samples were grouped based on the number of vaccine doses received by each study participant and descriptive statistics for the neutralising antibody titres in each group were generated in GraphPad Prism v8.4.3.

**Supplementary** **Table S2**: **Effect of number of vaccine doses on antibody titre, comparison between variants at each dose.** Antibody titres for each group were compared by One way ANOVA using Friedman's test, corrected for multiple comparisons with Dunn's test and comparing the mean rank of each column with every other column.

**Supplementary Table S3**: **Amino acid mutations associated with the SARS-CoV-2 variants.** Amino acid mutations associated with the variants investigated in the study (XBB.1.5, KP.3.1.1, LB.1 and XEC) the ancestral JN.1 variant, and the parents of the XEC recombinant (KP.3.3 and KS.1.1). Mutations are as accessed from cov-spectrum.org, 10th February, 2025 and are those mutations in the spike of ~100% of variants.

**Supplementary Table S4: Neutralising antibody titres against vaccine antigens and emerging variants, stratified by age group.** Samples were grouped based on the age of each study participant and descriptive statistics for the neutralising antibody titres in each group were generated in GraphPad Prism v8.4.3.

**Supplementary Table S5: Correlation between Age, Dose and Days since last vaccination.** The correlation between "Age", "Dose" and "Days since last vaccination" was assessed using a Spearman’s test (RStudio corr(data)). Anything >0.5 was considered highly correlated. "Age" and "Doses" had a strong positive correlation, while "Age" and "Days since last vaccination" had a strong negative correlation. A strong negative correlation was also evident between "Days since last vaccination" and "Doses".

**Supplementary Table S6: Relationship between IgG levels and vaccine type.** A GAM was used to model the non-linear relationship between predictors and IgG levels. Variables of significance included in the final GAM iteration included "Vaccine type", "Days since last vaccination", and the interactions between “Age" and "Doses” and “Doses and "Days since last vaccination”. The remaining variables were removed to avoid model overfitting. Estimate effect size shows the extent to which the variable influences IgG level (positive value for an increase, negative value for a decrease. Standard error (Std. Error) measures the variability in the estimate. Z-value assesses whether the variable has a meaningful effect on IgG (Estimate/Std. Error). P-value shows the statistical significance. In the NHSGGC study population, too few participants received the Moderna mRNA vaccination as their first dose to present a valid comparison with those having received the Astrazeneca vaccine as their first dose.

**Supplementary Table S7: Relationship between IgG levels and Age, Dose and Days since last vaccination.** A GAM was used to model the non-linear relationship between predictors and IgG levels. Variables of significance included in the final GAM iteration included "Vaccine type", "Days since last vaccination", and the interactions between “Age" and "Doses” and “Doses" and "Days since last vaccination”. The remaining variables were removed to avoid model overfitting. Estimated degrees of freedom indicates the flexibility of the smooth function (1 is a linear relationship, higher values represent increasing non-linearity). Reference degrees of freedom are the number of degrees of freedom set for testing. Chi-sq tests whether the smooth term explains variation in IgG levels (higher values for stronger effects). P-value shows the statistical significance.

**Supplementary Table S8: Relationship between IgG4 levels and vaccine type.** A GAM was used to model the non-linear relationship between predictors and IgG4 levels. Variables of significance included in the final GAM iteration included "Vaccine type", "Doses XBB.1.5" and "Days since last vaccination". The remaining variables were removed to avoid model overfitting. Estimate effect size shows the extent to which the variable influences IgG4 level (positive value for an increase, negative value for a decrease. Standard error (Std. Error) measures the variability in the estimate. Z-value assesses whether the variable has a meaningful effect on IgG (Estimate/Std. Error). P-value shows the statistical significance. In the NHSGGC study population, too few participants received the Moderna mRNA vaccination as their first dose to present a valid comparison with those having received the Astrazeneca vaccine as their first dose.

**Supplementary Table S9: Relationship between IgG4 levels and Days since last vaccination.** A GAM was used to model the non-linear relationship between predictors and IgG4 levels. Variables of significance included in the final GAM iteration included "Vaccine type", "Doses XBB.1.5" and "Days since last vaccination". The remaining variables were removed to avoid model overfitting. Estimated degrees of freedom indicates the flexibility of the smooth function (1 is a linear relationship, higher values represent increasing non-linearity). Reference degrees of freedom are the number of degrees of freedom set for testing. Chi-sq tests whether the smooth term explains variation in IgG4 levels (higher values for stronger effects). P-value shows the statistical significance.

**Supplementary Table S10. Relationship of neutralising antibody titre with viral variant and sex.** A GAM was used to model the non-linear relationship between predictors and neutralising antibody levels. Variables of significance included in the final GAM iteration included Variant, Sex, and the interactions between “Age, Doses” and “Doses, Days since last vaccination”. The remaining variables were removed to avoid model overfitting. Estimate effect size shows the extent to which the variable influences antibody titre (positive value for an increase, negative value for a decrease. Standard error (Std. Error) measures the variability in the estimate. Z-value assesses whether the variable has a meaningful effect on antibody titre (Estimate/Std. Error). P-value shows the statistical significance.

**Supplementary Table S11. Relationship between neutralising antibody titre and Age, Dose and Days since last vaccination.** A GAM was used to model the non-linear relationship between predictors and neutralising antibody levels. Variables of significance included in the final GAM iteration included Variant, Sex, and the interactions between “Age, Doses” and “Doses, Days since last vaccination”. The remaining variables were removed to avoid model overfitting. Estimated degrees of freedom indicates the flexibility of the smooth function (1 is a linear relationship, higher values represent increasing non-linearity). Reference degrees of freedom are the number of degrees of freedom set for testing. Chi-sq tests whether the smooth term explains variation in antibody titre levels (higher values for stronger effects). P-value shows the statistical significance.
